# Genome-wide association study and genomic prediction of resistance to stripe rust in current Central and Northern European winter wheat germplasm

**DOI:** 10.1007/s00122-022-04202-z

**Published:** 2022-08-26

**Authors:** Fahimeh Shahinnia, Manuel Geyer, Friederike Schürmann, Sabine Rudolphi, Josef Holzapfel, Hubert Kempf, Melanie Stadlmeier, Franziska Löschenberger, Laura Morales, Hermann Buerstmayr, Julio Isidro y Sánchez, Deniz Akdemir, Volker Mohler, Morten Lillemo, Lorenz Hartl

**Affiliations:** 1 Bavarian State Research Center for Agriculture, Institute for Crop Science and Plant Breeding, 85354 Freising, Germany; 2 SECOBRA Saatzucht GmbH, Lagesche Str. 250, 32657 Lemgo, Germany; 3 SECOBRA Saatzucht GmbH, Feldkirchen 3, 85368 Moosburg, Germany; 4 Saatzucht Donau GmbH & Co KG, 4981 Reichersberg, Mendelweg Austria; 5 Department of Agrobiotechnology, Institute of Biotechnology in Plant Production, University of Natural Resources and Life Sciences Vienna, Konrad-Lorenz-Straße 20, 3430 Tulln an der Donau, Austria; 6 Centro de Biotecnologia y Genómica de Plantas, Instituto Nacional de Investigación y Tecnologia Agraria y Alimentaria, Universidad Politécnica de Madrid, Campus de Montegancedo, Madrid, Spain; 7 Center for International Blood and Marrow Transplant Research (CIBMTR), National Marrow Donor Program/Be The Match, Minneapolis, MN USA; 8 Department of Plant Sciences, Norwegian University of Life Sciences, P.O. Box 5003, 1432 Ås, Norway

## Abstract

**Key message:**

We found two loci on chromosomes 2BS and 6AL that significantly contribute to stripe rust resistance in current European winter wheat germplasm.

**Abstract:**

Stripe or yellow rust, caused by the fungus *Puccinia striiformis* Westend f. sp. *tritici*, is one of the most destructive wheat diseases. Sustainable management of wheat stripe rust can be achieved through the deployment of rust resistant cultivars. To detect effective resistance loci for use in breeding programs, an association mapping panel of 230 winter wheat cultivars and breeding lines from Northern and Central Europe was employed. Genotyping with the Illumina® iSelect® 25 K Infinium® single nucleotide polymorphism (SNP) genotyping array yielded 8812 polymorphic markers. Structure analysis revealed two subpopulations with 92 Austrian breeding lines and cultivars, which were separated from the other 138 genotypes from Germany, Norway, Sweden, Denmark, Poland, and Switzerland. Genome-wide association study for adult plant stripe rust resistance identified 12 SNP markers on six wheat chromosomes which showed consistent effects over several testing environments. Among these, two marker loci on chromosomes 2BS (*RAC875_c1226_652*) and 6AL (*Tdurum_contig29607_413*) were highly predictive in three independent validation populations of 1065, 1001, and 175 breeding lines. Lines with the resistant haplotype at both loci were nearly free of stipe rust symptoms. By using mixed linear models with those markers as fixed effects, we could increase predictive ability in the three populations by 0.13–0.46 compared to a standard genomic best linear unbiased prediction approach. The obtained results facilitate an efficient selection for stripe rust resistance against the current pathogen population in the Northern and Central European winter wheat gene pool.

**Supplementary Information:**

The online version contains supplementary material available at 10.1007/s00122-022-04202-z.

## Introduction

Stripe or yellow rust is a major disease in bread wheat (*Triticum aestivum* L.) production under temperate climatic conditions or at high altitudes with a significant impact on grain yield and end-use quality characteristics (Singh et al. 2016). It is caused by *Puccinia striiformis* Westend f. sp. *tritici* (*Pst*), which is a biotrophic and heteroecious fungus. The pathogen is a common fungal disease of cereals and grasses, and various *Berberis* species can serve as alternate hosts (Hovmøller et al. 2011). Depending on various factors such as disease duration, infection stage, speed of disease development, susceptibility of cultivars, and favorable climatic conditions, yield reductions range from 10 to 40% (13% on average, Laidig et al. 2021) and can be as high as 100% if infection occurs at the seedling stage and persists until maturity (Afzal et al. 2007; Pradhan et al. 2020). Although stripe rust can be controlled with fungicides, economical, sustainable and environmentally sound management of this disease is needed. Breeding of resistant cultivars can be facilitated through identification, introduction, and subsequent selection of effective rust resistance genes during breeding cycles in wheat.

Fungal disease resistance genes in crop plants can generally be divided into all-stage resistance (ASR) genes and adult plant resistance (APR) genes. ASR genes are effective against avirulent pathotypes at all growth stages of the plant, are also referred to as race-specific, are inherited qualitatively, and can be overcome by new races. APR genes, on the other hand, express resistance only at post-seedling stages, are considered race-nonspecific, are inherited quantitatively, and tend to be durable (Bariana 2003; Rosewarne et al. 2013; Zetzsche et al. 2019). Because resistance levels in this group can be highly affected by temperature, it also includes high-temperature adult-plant (HTAP) resistance genes (Chen 2013). Since strong selection pressure is exerted on the pathogen to become virulent against a single ASR gene and thus survive, the use of a combination or pyramiding of APR genes with effective ASR genes is recommended to achieve commercially acceptable levels of resistance (Bariana 2003; Gessese et al. 2021).

The use of fungicides and the cultivation of resistant varieties have prevented devastating epidemics in Europe in the past, while genetic resistance is still the most effective and sustainable approach. In Europe, stripe rust occurred infrequently due to the use of a few key resistance genes with long-term efficacy such as *Yr1*, *Yr2*, *Yr3*, *Yr4*, *Yr6*, *Yr9*, *Yr15*, *Yr17*, *Yr25*, and *Yr32* (Hovmøller 2007; Hovmøller et al. 2016). In 2011, "Warrior," a new virulent stripe rust strain from the near-Himalayan region (Hovmøller et al. 2016), emerged simultaneously in several European countries and spread rapidly across much of the continent. According to monitoring by the Julius Kühn Institute (JKI, Germany), the *Pst* race "*Warrior* (-)" dominates the European yellow rust population, followed by the races "*Triticale2015*" and the original "*Warrior*," as well as a new race "*PstS15*," which was first discovered in 2020 (Flath et al. 2021). It seems that only a few resistance genes, including *Yr5*, *Yr10*, *Yr15*, and *Yr27*, are still effective against these races in European wheat (K. Flath 2022, JKI, personal communication).

The chromosomal positions of several stripe rust resistance genes and quantitative trait loci (QTL) were determined using classical mapping approaches (Tsomin et al. 1990; Bariana and McIntosh 1993; Michelmore et al. 1991; Xu et al. 2008; Edae et al. 2016; Gessese et al. 2021) and, more recently, using genome-wide association studies (GWAS) (Bouvet et al. 2021; Rollar et al. 2021). To complement traditional QTL mapping, GWAS combined with high-density SNP genotyping have been successfully used as powerful tools for discovery of stripe rust resistance loci in a global collection of winter wheat accessions (Bulli et al. 2016), spring wheat landraces (Kankwatsa et al. 2017), diverse Indian spring wheat cultivars (Kumar et al. 2020), US winter wheat cultivars and breeding lines (Mu et al. 2020), elite wheats of the International Maize and Wheat Improvement Center (CIMMYT) (Juliana et al. 2018), European winter wheat (Miedaner et al. 2019), and modern Chinese wheat (Jia et al. 2020).

Based on the “Catalogue of Gene Symbols for Wheat” available to date (https://wheat.pw.usda.gov/GG3/WGC), 85 formally named and more than 300 tentatively named genes or QTL located on different wheat chromosomes have been reported for stripe rust resistance (Maccaferri et al. 2015; Bulli et al. 2016; McIntosh et al. 2017; Mu et al. 2020). Of these, only a few genes, namely *Yr5/YrSP* and *Yr7* (Marchal et al. 2018), *Yr15* (Klymiuk et al. 2018), *Yr10* (Liu et al. 2014), *Yr18* (Krattinger et al. 2009), *Yr27* (Athiyannan et al. 2022), *Yr36* (Fu et al. 2009), *Yr46* (Moore et al. 2015), and *YrAS2388R* (Zhang et al. 2019) have been cloned and functionally characterized. Some chromosomes, such as 1B, 2A, 2B, and 7B, harbor a substantial number of genes or QTL that confer different types and degrees of resistance to stripe rust (Maccaferri et al. 2015). Although some race-nonspecific resistance genes are effective and durable against the new *Pst* races (Abou-Zeid and Mourad 2021), virulent *Pst* races have emerged against most *Yr* genes, rendering them ineffective (Maccaferri et al. 2015). Thus, continued efforts are needed to characterize new resistance sources for maintaining or increasing resistance levels. Compared to wild relatives of wheat, identification and characterization of resistance genes from landraces and cultivated genotypes is more preferable due to the absence of undesirable agronomic traits and chromosomal linkage drags in the latter (Burt et al. 2014; Gessese et al. 2021). The selection and combination of resistance genes already present in the advanced breeding pool allows for a faster development of new resistant varieties.

Classical phenotypic selection for *Pst* resistance is resource demanding, and its success strongly depends on environmental factors. The prediction of breeding values using molecular markers has become a promising approach to facilitate genomic selection. Marker-based prediction of breeding values can be categorized into marker-assisted selection (MAS), which makes use of a preselected set of markers associated with important resistance genes, and genomic selection (GS), which is based on genome-wide marker information. Previous studies have shown the potential of both MAS and GS for the prediction of *Pst* resistance, but comparisons of predictive abilities of the two approaches do not yet allow a clear conclusion about the optimal prediction method (Juliana et al. 2017; Muleta et al. 2017; Beukert et al. 2020b).

Compared to previous studies (Miedaner et al. 2019; Beukert et al. 2020a; Bouvet et al. 2021; Rollar et al. 2021) that used European winter wheat materials released in a broader time period, the present work identified QTL for stripe rust resistance in a collection of 230 current Central and Northern European winter wheat, examined the effects of these QTL in practical breeding programs and suggested prediction models for breeding cultivars with improved stripe rust resistance. Selection and combination of alleles already present in the advanced breeding pool is essential for improving durable resistance to stripe rust and for developing new wheat varieties. Specifically, the objectives of our study were to (1) perform GWAS in modern wheat germplasm using disease responses to current *Pst* populations assessed in field trials, (2) identify the sources of effective resistance alleles and associated QTL for use in breeding programs, (3) validate QTL in breeding materials, (4) compare the resistance loci identified in this study with previously reported *Yr* genes and QTL. Our results will help to understand the genetic basis of stripe rust resistance in Northern and Central European winter wheat and facilitate improvement of stripe rust resistance through MAS and GS.

## Materials and methods

### Plant materials and stripe rust assessment

With the aim to capture wide genetic variability across Europe, we selected a population of 230 winter wheat cultivars and breeding lines for the GWAS. Using the breeder´s knowledge and the coefficient of determination algorithm (Akdemir et al. 2021), we selected genotypes from Germany, Austria, Norway, Sweden, Denmark, Poland, and Switzerland comprising 157, 50, 14, 4, 3, 1, and 1 genotype(s), respectively (Table S1).

The 230 genotypes of the association panel were evaluated for stripe rust resistance at the adult plant stage in field experiments. Each entry was sown in two-row microplots 0.5–1.5 m long and 0.17–0.3 m wide with approximately 50 grains per row in October each year. Field trials were carried out at Lemgo and Lenglern in Germany, and in Tulln and Reichersberg in Austria in 2020 and 2021 (Table S2). We used non-replicated trials in favor of multiple sites, except for Tulln, where a randomized complete block trial with two replicates was conducted. To validate the results of this study, using same sowing conditions, two F_6_ populations of 1065 and 1001 German breeding lines from two consecutive breeding cycles were evaluated in Lemgo in 2020 and 2021, respectively, and another independent population of 175 breeding lines was evaluated in Lenglern in 2021. Field responses to either natural or artificial inoculation with *Pst* isolates (Table S1) were recorded between plant heading (Zadoks 50) and grain filling (Zadoks 80) stages when most flag leaves of susceptible controls had disease severity of at least 50%. Disease severity as a percentage of infected leaf area was scored 3–5 times in June using the modified Cobb scale (Peterson et al. 1948) and means for the recorded scores for stripe rust were used in the analyses.

### Statistical analysis

Implementation of the coefficient of determination algorithm for selection of the genotypes was done in R package "TrainSel" (R Core Team, 2017). After testing several transformation methods to meet the assumption of normality of residuals, the arcsine square root transformation method (Maccaferri et al. 2015) was chosen to prepare phenotypic data for association analysis. Analyses of variance (ANOVA) and estimation of adjusted means across environments were conducted using "PROC GLM" of the SAS statistical package v.9.4 (SAS Institute, Cary, USA) by applying the following model:

$$ y_{ij} = \mu + g_{i} + t_{j} + e_{ij} $$

where *y*_*ij*_ is the phenotypic value of genotype *i* in trial *j*, *µ* is the overall mean, *g*_*i*_ is the fixed effect of genotype *i*, *t* is the random effect of trial/environment *j* and *e*_*ij*_ is the random error, which was confounded with genotype × environment effects. The heritability coefficient was estimated according to the formula

$$ h^{2} = \frac{{\sigma_{{\text{G}}}^{2} }}{{\sigma_{{\text{G}}}^{2} + \frac{{\sigma_{{\text{e}}}^{2} }}{T}}} $$

where $\sigma_{{\text{G}}}^{2}$ is the genotypic variance, $\sigma_{{\text{e}}}^{2}$ is the residual variance, and *T* is the number of trials. Variance components for estimating *h*^2^ were derived from the above-mentioned model assuming a random genotypic effect.

### SNP genotyping

Genomic DNA of each genotype belonging to the association panel and validation populations was extracted from young leaf tissue according to the procedure of Plaschke et al. (1995). Genotyping was performed using the 25 K Infinium iSelect array (TraitGenetics, Seeland OT Gatersleben, Germany), which is an extension of 20 K array (Arif et al. 2021) plus 5000 Axiom array markers and candidate genes. SNP markers on the 25 K array were selected from the 90 K Infinium array, the 35 K Axiom wheat breeder array, and two proprietary TraitGenetics wheat arrays (135 K Axiom and 12 K Infinium). These markers were also selected based on the analysis of more than 2500 wheat cultivars, accessions and breeding lines according to the quality of performance on each analysis platform, their polymorphism information content (LD to flanking SNPs, MAF) and their position on the wheat reference genome (Dr. Jörg Plieske, TraitGenetics, personal communication). The physical position of the markers was determined by BLAST and e-value cut off for the highest probability match using the published genome sequence of Chinese Spring (IWGSC RefSeq v1.0). SNPs were filtered to avoid producing redundant genotyping information. The monomorphic SNPs and those with more than 10% missing values and minor allele frequency of less than 5% were excluded from further analysis using the "synbreed" package (Wimmer et al. 2012) in R (R Core Team 2017). Heterozygous marker signals were treated as missing data. Chromosomal positions of these SNPs were obtained from the 90 K consensus map (Wang et al. 2014).

### Population structure

The genetic structure of the 230 genotypes was determined using the Bayesian clustering program STRUCTURE (Pritchard et al. 2000). The output of STRUCTURE was analyzed in STRUCTURE HARVESTER (Earl and Vonholdt 2012) to determine the possible number of subpopulations using the ΔK ad hoc statistic. The best K value representing the optimal number of clusters in the populations was estimated as ΔK based on the rate of change of log-likelihood of data between successive values, as described by Evanno et al. (2005). Population structure was also analyzed by principal component analysis (PCA) to distinguish different groups using the "PROC PCA" in the SAS statistical package.

### Association mapping

Linkage disequilibrium (LD) between markers was estimated for the association mapping panel using observed versus expected allele frequencies in TASSEL 5.2.78 (Bradbury et al. 2007). LD decay was measured as the distance at which the average *R*^2^ value between pairwise SNPs fell to half its maximum value. Both Bayesian clustering method of STRUCTURE and PCA revealed a population structure in the panel. Marker-trait association in each environment and across environments were carried out using a mixed linear model that accounts for population structure (**Q**) and kinship matrix (**K**). The model can describe as follows:

$$ {\mathbf{y}} = {\mathbf{X\beta }}{ } + {\mathbf{Zu}}{ } + {\mathbf{e}}, $$

where **y** is the vector of observations, **β** is a vector containing fixed effects for genetic markers and population structure (**Q**), **u** is a vector of random additive genetic effects from multiple background QTL with **u** ~ N(0, $\sigma_{{\text{G}}}^{2}$ **K**), **X** and **Z** are the design matrices, and **e** is a vector of random residuals with **e** ~ N(0, $\sigma_{{\text{G}}}^{2}$ **I**). To provide the adjusted p-values, the false discovery rate (FDR) was calculated, using a threshold of < 5%, with the "q-value" package in R (R Core Team, 2017). To evaluate the performance of the models and appropriate thresholds, QQ plots drawn by TASSEL were inspected. Associations of SNP markers with stripe rust severity in each environment and across environments are represented as Manhattan plots. The physical position of significantly associated markers across environments was compared to previously published *Yr* genes and QTL using the “Catalogue of Gene Symbols for Wheat” (https://wheat.pw.usda.gov/GG3/WGC) and an integrated map for chromosomal positions of loci associated with reactions to *Pst* constructed by Bulli et al. (2016) and Maccaferri et al. (2015).

### Validation of associated markers

To detect allelic effect of associated SNP markers identified by GWAS in validation populations, the Student’s *t*-test was applied for evaluating statistically significant differences (*P* ≤ 0.05) between means of two allelic groups belonging to each locus of interest for disease severity.

### Putative candidate gene identification

Candidate genes for validated loci were identified by blasting the sequences of the markers on the corresponding chromosomes of the International Wheat Genome Sequencing Consortium RefSeq *v2.1* (https://urgi.versailles.inrae.fr/blast_iwgsc/?dbgroup=wheat_iwgsc_refseq_v2.1_chromosomes&program=blastn; Zhu et al., 2021) to retrieve the gene identifiers within a window of 2 Mb (1 Mb upstream and downstream) from the peak of each targeted marker. Gene ontology terms were obtained from EnsmblPlants using the biomaRt package (Durinck et al. 2009).

### Prediction of stripe rust resistance

The predictive ability of different linear regression models was evaluated using adjusted phenotypic mean values after arcsine square root transformation as response variable. Only a common set of SNP markers that remained after filtering in the GWAS panel and the three validation panels was considered for prediction. Linear regression using the ordinary least squares (OLS) method was performed using the "stats" package (R Core Team 2017). Following the matrix notation of the GWAS model shown above, the OLS model was fitted considering only fixed marker effects (**β**) and a random error (**e**). Genomic best linear unbiased prediction (GBLUP) models were fitted with rrBLUP (Endelman 2011) applying a model similar to the GWAS model, with the only exception of **β**, which only contained fixed marker effects without effects of population structure. To avoid collinearity due to linkage among detected markers, we discarded markers with a variance inflation factor > 5, from the design matrix **X** using the R package “car” (Fox and Weisberg 2019). Predictive ability was defined as the correlation between estimated breeding values and observed phenotypic values. Predictive ability was first estimated within the GWAS panel using a five-fold cross-validation with 200 replications (*N*_training_ = 184, *N*_test_ = 46). The predictive ability of these models was also evaluated in the three independent validation populations using the complete GWAS panel for model training.

## Results

### Phenotypic evaluation for stripe rust

Depending on the environmental conditions and the *Pst* pathotypes present in the different field trials, a wide variation was observed for the severity of stripe rust in the panel in each environment (ranging from 0 to 87.5%). The four breeding lines TS085, TS175, TS185, and TS220 and three cultivars TS140 (Gentleman), TS173 (Tobak) and TS195 (Sinatra) from Germany and the cultivar TS027 (Mariboss) from Norway were completely resistant in different field trials and across environments (Table S1). The cultivars TS012 (Rida) from Norway and TS014 (Akteur) from Germany were highly susceptible in this panel (Table S1). Stripe rust severity was left skewed in each experiment and for means across experiments (Table 1; Fig. S1.A). Significant (*P* < 0.05) and positive correlations ranging from 0.57 to 0.76 were observed between all pairs of test environments (Table 2), with the highest correlation (0.76) between Lemgo 20 and Lemgo 21, while the lowest correlation (0.57) was found between Lenglern 21 and Reichersberg 21.. Normality of residuals was achieved by applying the arcsine square root transformation method (Fig. S1.B). ANOVA revealed significant differences (*P* < 0.01) among the genotypes. The estimate of genetic variance ($\sigma_{{\text{G}}}^{2}$ = 0.026) contributed to a high broad-sense heritability for stripe rust resistance (*h*^2^ = 0.87; $\sigma_{{\text{T}}}^{2}$ = 0.017; $\sigma_{{\text{e}}}^{2}$ = 0.019).

**Table 1:** Minimum (Min), median, average (Mean), maximum (Max), and standard error (SE) of stripe rust disease severity (%) in the 230 lines of the genome-wide association study (GWAS) panel

Field trial	Min	Median	Mean	Max	SE
Lemgo 20	0	13.7	19.4	87.5	1.4
Lemgo 21	0	5	9.1	65	0.7
Tulln 21	0	5	7.8	50	0.5
Reichersberg 21	0	16.2	20.4	80	1.1
Lenglern 21	0	1	3.6	50	0.4
Across environments	0	9.1	11.7	51	0.7

The number in the name of each field trial indicates the year of phenotypic evaluation

**Table 2:**
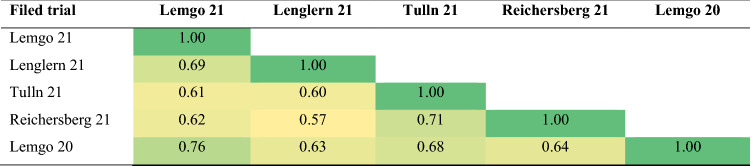
Phenotypic correlations of stripe rust severity scores (based on mean values) of the 230 lines in the GWAS panel among field trials

### Genotyping, population structure and LD decay

After marker filtering, 8812 informative and polymorphic SNP markers (Table S3) with an average minor allele frequency of 0.26 were used for analyzing population structure, LD, and genetic association. Of these, 3390, 3977, and 1445 markers belonged to subgenomes A, B, and D, respectively, with most markers (683) found on chromosome 5B and the fewest markers (70) on chromosome 4D. All SNPs were physically anchored to the reference sequence of Chinese Spring wheat.

**Fig. 1:**
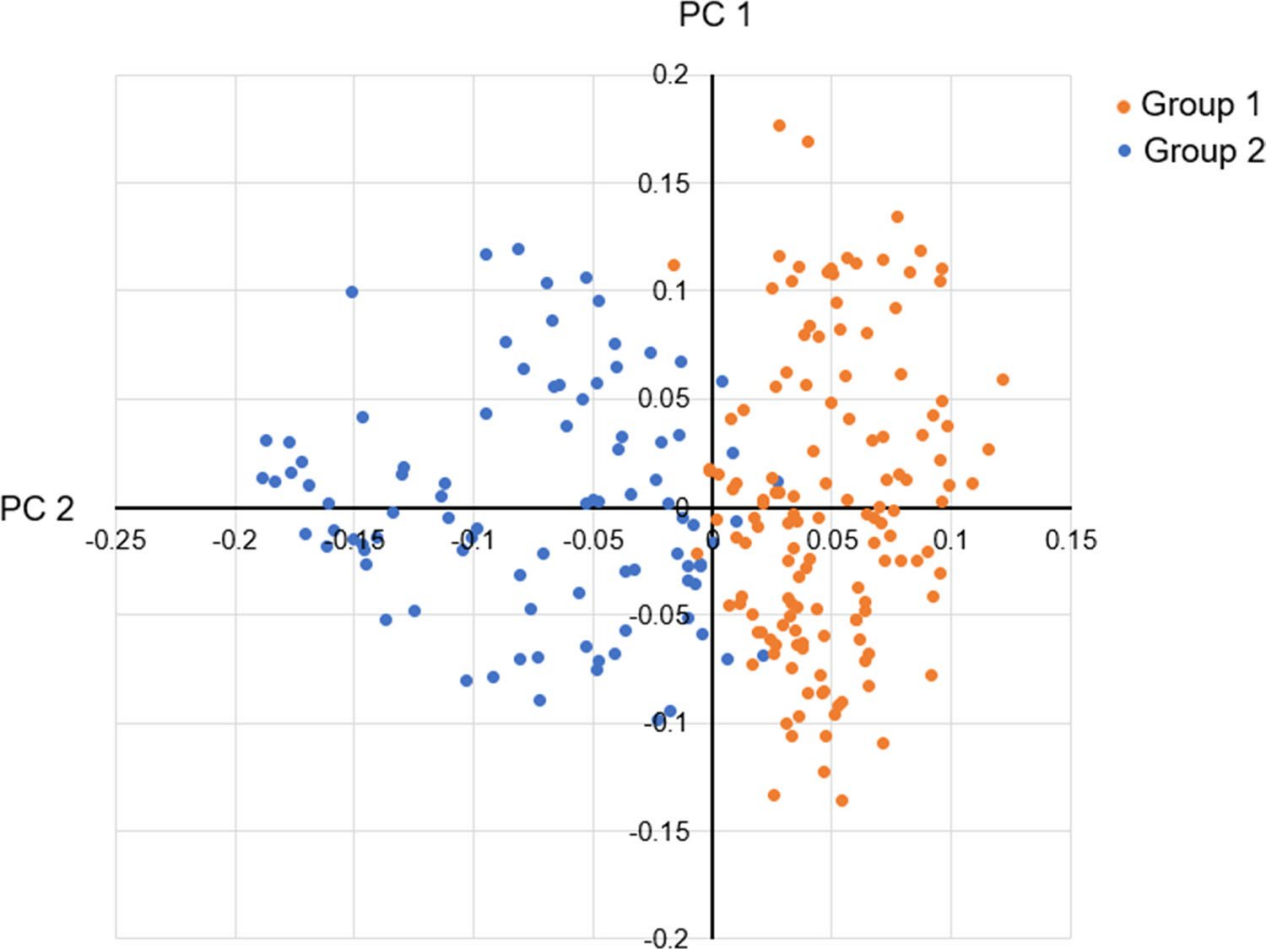
Principal component analysis showing two groups corresponding to two subpopulations in STRUCTURE analysis. Group 1 consisted of 92 Austrian breeding lines and cultivars, which were separated from the other 138 genotypes from Germany, Norway, Sweden, Denmark, Poland, and Switzerland (Group 2)

Two subpopulations were detected in the winter wheat panel of 230 genotypes (Fig. S2). The number of subpopulations (**K**) was estimated based on the rate of change of the log-likelihood of the data between successive **K** values. In the plot of K versus ΔK, a reduction in the slope was observed at ΔK = 2 (Fig. S2A). Therefore, the panel was divided into two subgroups based on the corresponding population membership coefficients (**Q**) of the individuals, with subgroup 1 containing 92 genotypes mainly from Austria and subgroup 2 containing 138 genotypes mainly from Germany Norway, Sweden, Denmark, Poland, and Switzerland (Fig. S2B). PCA also classified the panel into two subpopulations (Fig. 1). PC1 and PC2 accounted for 20% and 10% of the total marker-based variation, respectively. In the LD analysis (Fig. S3), patterns between significantly associated markers were determined using the squared Pearson correlation coefficient (*R*^2^) between SNP markers as a function of physical map position between markers. As shown in the scatter plot (Fig. S3), the strength of LD due to linkage decreases as the physical distance between SNPs in the genome increases. LD decay reached *R*^2^ = 0.2 at 200 Mb.

### GWAS and candidate genes for stripe rust resistance

GWAS using a mixed linear model identified 67 different loci on 12 wheat chromosomes, namely 1A, 2A, 2B, 3A, 3B, 4B, 5B, 5D, 6A, 7A, 7B, and 7D which were significantly associated with stripe rust disease severity assessed in at least one trial (Table 3; Fig. S2). The QQ plots evaluating the performance of the mixed linear models indicated a high corrective effect of the GWAS model (Fig. S4). The percentage of explained phenotypic variance (*R*^2^) of the associated markers ranged from 5 to 11%, while the effect size of those markers ranged from − 0.37 to 0.28 (Table 3). The strongest association (*R*^2^ = 11%, *p* value = 0.0001) was found for the SNP markers *Jagger_c1423_102* and *GENE-4167_145* on chromosome 6A in Lemgo 21 (Table S4). Resistant allele G at marker locus *IAAV1743* and susceptible allele G at marker locus *RAC875_c1226_652* both on chromosome 2B showed the maximum effects (0.37 and 0.28, respectively) on stripe rust severity identified in Lemgo 20 and Reichersberg 21 field trials, respectively (Table S4). Of the 67 markers, 12 SNP markers on chromosomes 2B, 4B, and 6A, 7A, 7B, and 7D displayed significant associations for means across environments (Table 4). Four SNP markers on chromosomes 2B (*Jagger_c6853_60*), 7A (*BS00093016_51*), 7B (*AX-95154562*), and 7D (*AX-94720261*) were significantly associated solely with the average stripe rust severity across all field trials (Table 3). In addition, eight SNP markers were found to be common (Table S3; Table 4) in at least two environments on chromosomes 2B (*RAC875_c1226_652*, *IAAV1743*, *Ra_c6266_136*), 4B (*AX-94684920*), and 6A (*Tdurum_contig29607_413*, *Jagger_c1423_102*, *GENE-4167_145*, *BS00040814_51*).

**Table 3:** Number of SNPs, chromosomal locations and range of marker effect on disease severity and *R*^2^ (phenotypic variance explained) for associated markers with stripe rust severity identified through GWAS in the winter wheat diversity panel evaluated in different field trials and across environment

Field trial	No. of SNPs	Chromosome	Effect	*R*^2^ (%)
Lemgo 20	14	1A, 2B, 3A, 3B, 4B,6A	− 0.37 to 0.25	5–7
Lemgo 21	13	2B, 6A, 7A	− 0.19 to 0.25	6–11
Tulln 21	23	2A, 2B, 7A, 7B, 7D	− 0.16 to 0.07	6–7
Reichersberg 21	10	1A, 4B, 5B, 5D, 7A, 7B	− 0.32 to 0.28	5–6
Lenglern 21	6	2B, 4B, 5A, 7A	− 0.13 to 0.12	5–7
Across environments	12	2B, 4B, 6A, 7A, 7B, 7D	− 0.10 to 0.15	5–7

**Table 4:** SNP markers associated with stripe rust severity in the winter wheat panel evaluated at the adult plant stage for the transformed means across environments. The marker alleles associated with increased resistance are bolded

Marker	Chromosome	Position (bp)	*R*^2^ (%)	Allele	Effect	*P* value
*RAC875_c1226_652*	2B	157,693,607	0.06	A/**G**	0.15	0.0004
*IAAV1743*	2B	439,225,308	0.06	**G**/T	− 0.22	0.0002
*Ra_c6266_136*	2B	440,214,889	0.06	**A**/G	− 0.22	0.0003
*Jagger_c6853_60*	2B	547,058,598	0.06	A/**G**	0.20	0.0004
*RFL_Contig4718_1269*	2B	553,623,396	0.05	**A**/G	− 0.10	0.0006
*AX-94684920*	4B	581,078,314	0.06	**C**/T	− 0.11	0.0003
*Tdurum_contig29607_413*	6A	609,380,034	0.08	C/**T**	0.15	0.0000
*Jagger_c1423_102*	6A	611,326,235	0.05	**A**/G	− 0.11	0.0006
*GENE-4167_145*	6A	611,328,899	0.06	**C**/T	− 0.12	0.0003
*BS00093016_51*	7A	515,199,467	0.05	**A**/C	− 0.10	0.0010
*AX-95154562*	7B	686,650,881	0.07	**A**/T	− 0.15	0.0002
*AX-94720261*	7D	414,283,385	0.05	**A**/G	− 0.07	0.0010

**Fig. 2:**
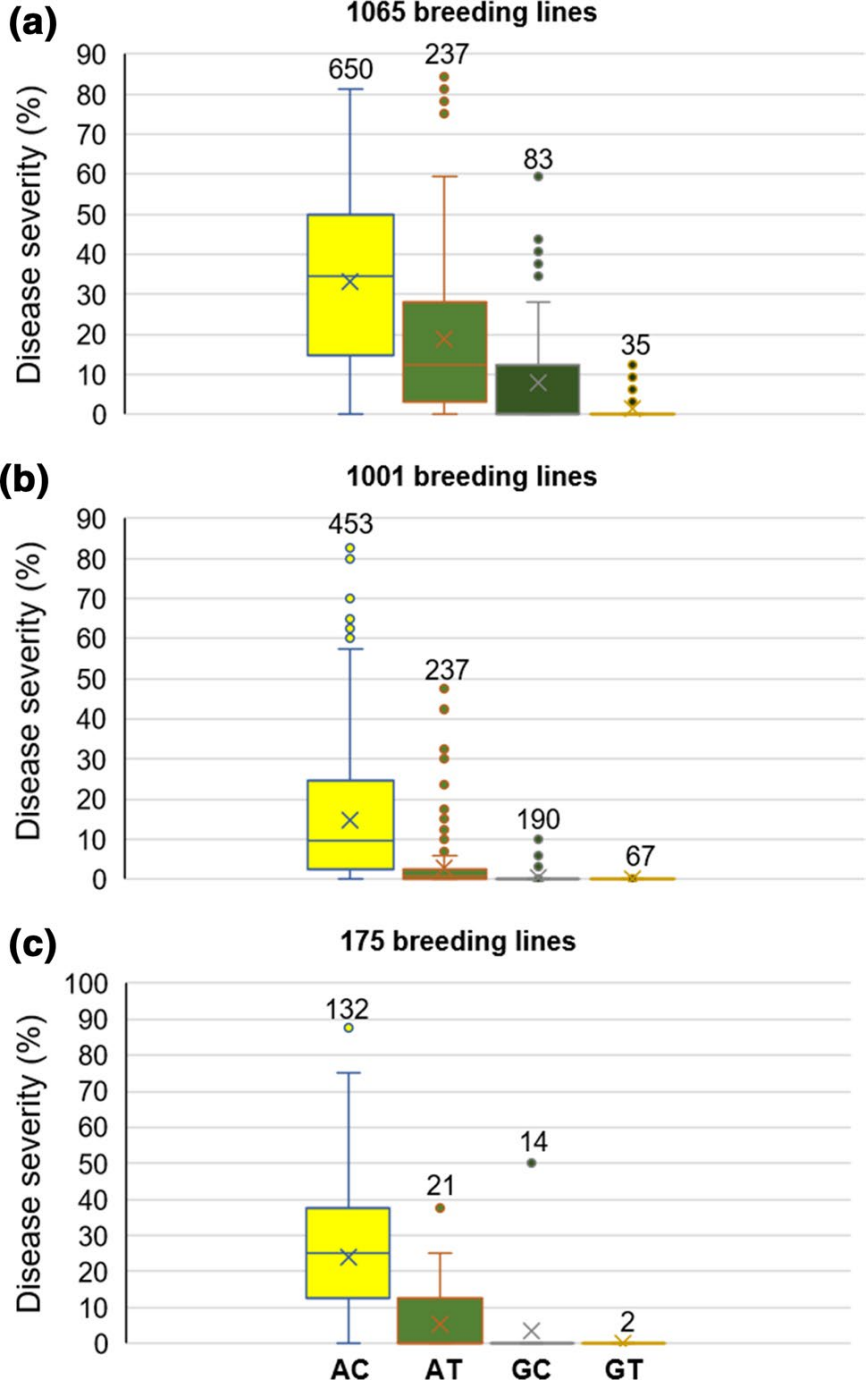
Effects of allelic combination of the markers located on chromosomes 2B (*RAC875_c1226_652*, A and G alleles) and 6A (*Tdurum_contig29607_413*, C and T alleles) on disease severity (%) in validation populations of **a** 1065 and **b** 1001 breeding lines evaluated in Lemgo in 2020 and 2021, respectively, and **c** 175 breeding lines evaluated in Lenglern in 2021. The more susceptible alleles are shown in yellow. The number of lines in each group is presented at the top of each box plot

Notably, the association of only two markers, namely *RAC875_c1226_652* on chromosome 2B and *Tdurum_contig29607_413* on chromosome 6A, with stripe rust resistance were validated by Student’s *t*-test (*P* ≤ 0.001) in three validation populations of 1065, 1001, and 175 breeding lines assessed for disease severity in Lemgo, Germany, in 2020 and 2021 and in Lenglern, Germany, in 2021. For the marker *RAC875_c1226_652*, the G allele and for the marker *Tdurum_contig29607_413*, the T allele contributed to a 10–18% and 8–17% reduction in stripe rust disease severity in the validation populations, respectively (Fig. 2). Lines in the validation that harbored the resistance improving alleles at both markers (Fig. 2), showed a highly to completely resistant phenotype (0–1.6% stripe rust severity) compared to haplotypes with the “susceptible” alleles (10–35% stripe rust severity). Interestingly, only one breeding line (TS128) in the GWAS panel possessed this allele combination.

To further investigate the validated loci, sequences of the two SNP markers *RAC875_c1226_652* and *Tdurum_contig29607_413* were aligned to the physical map of the reference genome to search for annotated genes. As a result, 20 to 68 putative candidate genes (Table S5) were identified in a 2-Mb physical interval around the peak markers on chromosomes 2B and 6A, respectively. The genes *TraesCS2B02G182800* and *TraesCS6A02G399600*, which were directly tagged by these markers, encode a putative disease resistance protein RGA4 (LOC123044110), BST_chr2B_nlr_143 and the E3 ubiquitin protein ligase RHB1A (LOC123129052), respectively, and are known to be involved in disease resistance and defense mechanisms in wheat.

Finally, the corresponding physical interval of the associated loci for means across environments identified in the present study were compared with previously reported *Yr* genes and QTL (Table S6). The physical interval of the compared QTL showed overlap with eighteen QTL and six genes including *Yr27*, *Yr5*, *Yr44*, *YrSp*, *Yr62*, and *Yr18.*

### Predicting resistance to stripe rust

The potential of MAS and GS to select for *Pst* resistant wheat was evaluated using (1) 6846 genome-wide SNP markers that were common between all four data sets, (2) the set of 12 markers identified in the present GWAS based on transformed means across environments, and (3) the two associated markers that were also significant in the three validation panels. Two of the 12 markers (*Ra_c6266_136* and *GENE-4167_145*) were excluded from all fixed effect matrices to avoid collinearity resulting from linkage. Five-fold cross-validation of ordinary least squares (OLS) and GBLUP models within the GWAS panel confirmed the association between the remaining ten markers and *Pst* resistance, whereas a reduced model with only *RAC875_c1226_652* and *Tdurum_contig29607_413* as predictors resulted in a lower predictive ability (Table 5). In this population, the highest predictive ability was achieved using a GBLUP model with all ten detected markers included as fixed effects. Predicting breeding values in three independent validation panels showed that OLS and GBLUP including the QTL-linked markers as covariates outperformed the standard GBLUP model. Contrary to the cross-validation within the GWAS data set, the estimated breeding values of the three validation panels were more accurate with only *RAC875_c1226_652* and *Tdurum_contig29607_413* as fixed effects compared to models including all ten associated markers as predictors. In these independent data sets, the highest predictive ability was achieved using either OLS or GBLUP treating the two above-mentioned markers as fixed effects. With these models, we could increase predictive ability in the three populations by 0.13–0.46 compared to the standard GBLUP approach.

**Table 5:** Predictive ability of ordinary least square (OLS) and genomic best linear unbiased prediction (GBLUP) models in four data sets

Method	GWAS panel	1065 breeding lines	1001 breeding lines	175 breeding lines
OLS (10 QTL-linked SNPs)	0.53 ± 2.8 10^−3^	0.34	0.44	0.26
OLS (2 QTL-linked SNPs)	0.40 ± 3.7 10^−3^	0.46	0.59	0.44
GBLUP	0.45 ± 3.4 10^−3^	0.33	0.40	− 0.01
GBLUP + 10 QTL-linked SNPs	0.64 ± 2.2 10^−3^	0.42	0.49	0.24
GBLUP + 2 QTL-linked SNPs	0.51 ± 3.5 10^−3^	0.49	0.59	0.45

Predictive ability in the GWAS panel was obtained by fivefold cross-validation. Breeding values of three independent validation populations were estimated using the GWAS panel for model training

## Discussion

Host plant resistance is generally the most ecological and economic strategy for stripe rust disease control. Integration of additional resistance alleles into the genetic background of already resistant cultivars is important for improving the durability of stripe rust resistance in wheat breeding. Understanding the genetic basis of stripe rust resistance could facilitate the transfer of existing or new resistance alleles into high-yielding and regionally adapted bread wheat lines.

In the present study, the frequency distribution of the percentage of disease severity assessed in the field trials was right skewed with a large proportion of resistant lines (Fig. S1.A), suggesting that many effective APR and possibly race-nonspecific alleles may be present in this panel. Similar results were obtained when the disease severity of stripe rust was assessed in a European winter wheat diversity panel of 158 old and new wheat cultivars (Miedaner et al. 2020) and in 419 pre-breeding lines developed at CIMMYT (Ledesma-Ramírez et al. 2019). All these studies indicated that this type of resistance is of high priority in breeding programs: it is established through the targeted crossing of resistant lines followed by high selection pressure in early generations. In addition, the broad-sense heritability coefficient (*h*^2^ = 0.87) obtained in this study suggests that phenotypic variation in stripe rust severity was mainly due to genotypic effects. High broad-sense heritability values have been reported in previous studies by Ling et al. (2012), Beukert et al. (2020a), and Abou-Zeid and Mourad (2021).

GWAS identified 67 QTL for stripe rust resistance in a panel of 230 winter wheat cultivars and breeding lines. Among all marker loci identified in five environments, only eight SNPs were found significant in at least two environments, therefore could be considered as stable QTL. Four SNPs were found significantly associated with stripe rust resistance for means across environments, but not in individual environments. Among these, two QTL appeared particularly attractive for stripe rust resistance breeding, namely those predicted by markers *RAC875_c1226_652* (chromosome 2B) and *Tdurum_contig29607_413* (chromosome 6A). Lines in the validation populations combining the favorable alleles at these loci displayed a near immune phenotype.

The results of the present study revealed QTL close to the genomic regions of the stripe rust resistance genes *Yr5/YrSP* (Macer 1966; Murphy et al. 2009; Feng et al. 2015) and *Yr44* (Sui et al. 2009) on chromosome 2B, *Yr62* (Lu et al. 2014) on chromosome 4B, and *Yr18* (Singh 1992) on chromosome 7D near the SNP markers *IAAV1743*, *AX-94684920*, and *AX-94720261*, respectively. *Yr5* provides a high level of resistance to stripe rust and originates from spring spelt *Triticum spelta* var. *album* (Macer 1966). It is allelic to *YrSP* and paralogous to *Yr7*, both of which have been overcome by several *Pst* isolates. A QTL (*qYr.A*) in the same genomic region was previously reported by Losert et al. (2017) in a diverse set of 919 triticale genotypes from the private and public breeding sectors in Europe. In this region, five QTL controlling stripe rust resistance (Table S6) have been reported in common wheat (Guo et al. 2008; Jighly et al. 2015; Ando et al. 2018; Ren et al. 2012; Tehseen et al. 2021).

We found a QTL on chromosome 4B in the region of the *Yr62* gene. Lu et al. (2014) found a QTL on chromosome 4BL (*QYrPI192252.wgp*-*4BL*) in a mapping population of 150 F_5_ recombinant inbred lines (derived from a cross between PI 192252 and “Avocet susceptible”) which explained 40–60% of the total phenotypic variation of the relative area under the stripe rust disease progress curve and was inherited as a single gene. The gene, named *Yr62*, provides a high level of HTAP resistance and was located proximal to *Yr50*, transferred from *T. intermedium* into wheat. Jia et al. (2020) and Naruoka et al. (2015) also reported two QTL for stripe resistance in a similar region on chromosome 4BL.

We discovered a QTL in the *Lr34/Yr18* region on chromosome 7DS. Cloning of *Lr34/Yr18* has shown that the gene encodes an adenosine triphosphate-binding cassette transporter that resembles a pleiotropic drug resistance transporter (Krattinger et al. 2009). *Lr34/Yr18* is active at the adult plant stage and shows moderate but durable resistance to stripe and leaf rust (Spielmeyer et al. 2005). It appears that the gene is relatively common in German cultivars (Zetzsche et al. 2020). However, further investigations are needed to confirm whether the QTL identified in the present study on chromosomes 2BL, 4BL, and 7DS are allelic to or distinct from *Yr5/YrSP*, *Yr44*, *Yr62*, and *Yr18*, respectively.

Our study also detected four loci associated with stripe rust resistance on chromosomes 2BS, 6AL, 7AL, and 7BL (Table 4), which could correspond to QTL previously reported by Prins et al. (2011), Vazquez et al. (2012), Rosewarne et al. (2012), Agenbag et al. (2014), Miedaner et al. (2019), Beukert et al. (2020a), Jia et al. (2020), and Rollar et al. (2021). Of these, QTL on chromosomes 2BS and 6AL were identified in German plant materials including a winter wheat diversity panel (Miedaner et al. 2019), a hybrid wheat panel (Beukert et al. 2020a), and a multiparental population (Rollar et al. 2021). Interestingly, these two QTL were not reported in the worldwide collections of hexaploid spring (Maccaferri et al. 2015) and winter wheat (Bulli et al. 2016), suggesting that they were specifically enriched through European breeding activities.

On chromosome 2B, the putative QTL associated with *RAC875_c1226_652* was in a region that referred to BST_chr2B_nlr_143 and disease resistance protein RGA4. In this region, a QTL (*QYr.sgi-2B.1*) was previously mapped by Agenbag et al. (2014) near the marker *IWB52095*, located at 157.694 Mbp of the wheat physical map (IWGSC Refseq v.1). More recently, the QTL was cloned in bread wheat as a major factor for the race-specific disease resistance gene *Yr27*, which encodes an intracellular immune receptor (Athiyannan et al. 2022). *Yr27* is allelic to leaf rust resistance gene *Lr13* with 97% sequence identity. The predicted coding sequence of the gene with a length of 3219 base pairs encodes a protein of 1072 amino acids with an N-terminal coiled-coil domain, a central NB-ARC domain, and a carboxy-terminal leucine-rich repeat (LRR) domain (Athiyannan et al. 2022). In rice, resistance to *Magnaporthe oryzae* is mediated by a pair of interacting nucleotide-binding site leucine-rich repeat domain-containing immune sensors, RGA4 and RGA5 (Césari et al. 2014). RGA4 mediates cell death but is repressed by RGA5. The repressor is neutralized by binding pathogen-derived proteins to the dimer. RGA4 and RGA5 interact through their CC domains to form homo- and heterocomplexes. In addition, BLAST searches using the sequence of another SNP marker on chromosome 6AL (*Tdurum_contig29607_413*) yielded direct hits for a gene annotated as E3 ubiquitin ligase protein, which is a module that controls innate immunity and programmed cell death in plants and strongly contributes to promoting antimicrobial defense while preventing autoimmunity (You et al. 2016). However, the mechanisms contributing to this immune homeostasis are poorly understood. Understanding the mode of action between candidate genes and their effects on disease resistance can help in the development of functional and predictive markers to detect resistant. It also provides new insights into the genetic mechanisms controlling stripe rust resistance in wheat and lays the foundation for characterization, cloning, and manipulation of genes in future studies. Since most of the genes/QTL identified in this study are already present in adapted varieties and/or elite breeding lines, pyramiding of these genes for breeding new resistant varieties seems relatively straightforward.

The markers identified in the present study showed great potential to facilitate an efficient selection for *Pst* resistance. OLS and GBLUP models that included *RAC875_c1226_652* and *Tdurum_contig29607_413* as fixed effects allowed a more accurate prediction of breeding values in the three independent panels compared to a GBLUP model with only the intercept as fixed effect. More complex models with all ten QTL-linked markers as fixed effects were only superior within the GWAS panel but yielded comparably low predictive abilities in the independent validation panels, which can be explained by overfitting. The value of preselected markers for the prediction of *Pst* resistance was evaluated in previous studies, but the comparison with genome-wide markers did not yield consistent results (Juliana et al. 2017; Muleta et al. 2017; Beukert et al. 2020b). These differing outcomes could be attributed to the size and composition of the training set, the genetic architecture of *Pst* resistance in the analyzed populations, and the prevalent pathotypes. Nevertheless, the present study demonstrates that, for the Northern and Central European winter wheat gene pool and current pathogen races, the validated QTL on chromosomes 2B and 6A and associated markers appear highly attractive to facilitate selection of *Pst* resistant cultivars. Since GBLUP with fixed effects for *RAC875_c1226_652* and *Tdurum_contig29607_413* was superior to OLS, we recommend including these markers in GS models to enrich quantitative, minor-effect alleles for a more sustainable resistance in combination with large-effect alleles.

Overall, our study identified resistant genotypes and the potential source of effective resistance alleles and associated QTL that could be used to improve stripe rust resistance levels in the current Northern and Central European breeding materials, particularly through genomic approaches.

## Supplementary Information

Below is the link to the electronic supplementary material.

Table S1 List of 230 winter wheat cultivars and breeding lines assembled from the Northern and Central European countries Germany, Austria, Norway, Sweden, Denmark, Poland, and Switzerland. Mean values of stripe rust scores (%) evaluated in field trials and SNP alleles at 12 QTL identified across environments are presented. Table S2 Information of experiments conducted in field trials for evaluating stripe rust in 230 association panel. Table S3 List of 8812 SNP markers and their chromosomal position used for GWAS analysis in the association panel of 230 genotypes. Table S4 SNP markers identified in the winter wheat panel associated with stripe rust responses were evaluated in different field trials and across environments during 2020–2021. The marker alleles associated with increased resistance are bolded. Table S5 Functional annotation of putative candidate genes within 2 Mb (1 Mb upstream and downstream) physical interval of the peaks of validated markers associated with stripe rust disease resistance on chromosomes 2B (*RAC875_c1226_652*) and 6A (*Tdurum_contig29607_413*). The genes indicated in bold were tagged directly by the markers. Table S6 Comparison between QTL identified in the present study based on means across environments and previously reported genes and QTL controlling stripe rust resistance in common wheat. (XLSX 7581 kb)

Fig. S1. a) Frequency distribution of stripe rust disease severity (%) in different field trials and across environments.(TIF 900 kb)

b) Residual frequency distribution of arcsine square root transformed of disease severity in different field trials and across environments. (TIF 1164 kb)

Fig. S2 Population structure among 230 winter wheat cultivars and breeding lines. a) Determination of the number of subpopulations via the ad hoc statistic ΔK. a) Stacked bar plots of ancestry relationship of genotypes based on membership coefficient of individuals (Q). (TIF 88 kb)

Fig. S3 Scatter plot of linkage disequilibrium (R^2^) versus inter-marker physical distance (Mbp), with the average R^2^ of increasing intervals of 0.5 Mbp physical distance plotted as a red line. (TIF 145 kb)

Fig. S4. Manhattan plots showing the association of single nucleotide polymorphisms in the 230 genotypes (left) and quantile–quantile plot comparing the performance of the mixed linear model (right) used in the genome-wide association study for stripe rust resistance in different field trials (a-e) and across environments (f). The horizontal black line represents the genome-wide significance threshold. (TIF 1586 kb)

## Data Availability

All datasets generated for this study are included in the article/Supplementary Material.

## Abbreviations

ANOVA: Analysis of variance
APR: Adult plant resistance
ASR: All-stage resistance
CIMMYT: International Maize and Wheat Improvement Center
FDR: False discovery rate
GBLUP: Genomic best linear unbiased prediction
GS: Genomic selection
GWAS: Genome-wide association study
HTAP: High-temperature adult-plant
LD: Linkage disequilibrium
LRR: Leucine-rich repeat
MAS: Marker-assisted selection
OLS: Ordinary least squares
PCA: Principle component analysis
*Pst*: *Puccinia striiformis*
QTL: Quantitative trait loci
SNP: Single nucleotide polymorphism

## References

[CR1] Abou-Zeid MA, Mourad AMI (2021). Genomic regions associated with stripe rust resistance against the Egyptian race revealed by genome-wide association study. BMC Plant Biol.

[CR2] Afzal SN, Haque MI, Ahmedani MS, Bashir S, Rattu AR (2007). Assessment of yield losses caused by *Puccinia striiformis* triggering stripe rust in the most common wheat varieties. Pak J Bot.

[CR3] Agenbag GM, Pretorius ZA, Boyd LA, Bender CM, MacCormack R (2014). High-resolution mapping and new marker development for adult plant stripe rust resistance QTL in the wheat cultivar Kariega. Mol Breed.

[CR4] Akdemir D, Rio S, Isidro y Sánchez J (2021) TrainSel: an R package for selection of training populations. Front Genet 12:655287. 10.3389/fgene.2021.655287 10.3389/fgene.2021.655287 PMC8138169 34025720

[CR5] Ando K, Rynearson S, Muleta KT, Gedamu J, Girma B (2018). Genome-wide associations for multiple pest resistances in a Northwestern United States elite spring wheat panel. PLoS ONE.

[CR6] Arif MAR, Shokat S, Plieske J, Ganal M, Lohwasser U (2021). A SNP-based genetic dissection of versatile traits in bread wheat (*Triticum aestivum* L.). Plant J.

[CR7] Athiyannan N, Abrouk M, Boshoff WHP, Cauet S, Rodde N (2022). Long-read genome sequencing of bread wheat facilitates disease resistance gene cloning. Nat Genet.

[CR8] Bariana HS, Murray BG, Murphy DJ (2003). Breeding for disease resistance. Encyclopedia of applied plant sciences.

[CR9] Bariana HS, McIntosh RA (1993). Cytogenetic studies in wheat. XV. Location of rust resistance genes in VPM1 and their genetic linkage with other disease resistance genes in chromosome 2A. Genome.

[CR10] Beukert U, Liu G, Thorwarth P, Boeven PHG, Longin CFH (2020). The potential of hybrid breeding to enhance leaf rust and stripe rust resistance in wheat. Theor Appl Genet.

[CR11] Beukert U, Thorwarth P, Zhao Y, Longin CFH, Serfling A (2020). Comparing the potential of marker-assisted selection and genomic prediction for improving rust resistance in hybrid wheat. Front Plant Sci.

[CR12] Bouvet L, Percival-Alwyn L, Berry S, Fenwick P, Campos Mantello C (2021). Wheat genetic loci conferring resistance to stripe rust in the face of genetically diverse races of the fungus *Puccinia striiformis* f. sp. *tritici*. Theor Appl Genet.

[CR13] Bradbury PJ, Zhang Z, Kroon DE, Casstevens TM, Ramdoss Y (2007). TASSEL: software for association mapping of complex traits in diverse samples. Bioinformatics.

[CR14] Bulli P, Zhang J, Chao S, Chen X, Pumphrey M (2016). Genetic architecture of resistance to stripe rust in a global winter wheat germplasm collection. G3.

[CR15] Burt C, Griffe LL, Ridolfini AP, Orford S, Griffiths S (2014). Mining the Watkins collection of wheat landraces for novel sources of eyespot resistance. Plant Pathol.

[CR16] Césari S, Kanzaki H, Fujiwara T, Bernoux M, Chalvon V (2014). The NB-LRR proteins RGA4 and RGA5 interact functionally and physically to confer disease resistance. EMBO J.

[CR17] Chen XM (2013). High-temperature adult-plant resistance, key for sustainable control of stripe rust. Am J Plant Sci.

[CR18] Durinck S, Spellman PT, Birney E, Huber W (2009). Mapping identifiers for the integration of genomic datasets with the R/Bioconductor package. Nat Protocols.

[CR19] Earl DA, Vonholdt BM (2012). STRUCTURE HARVESTER: a website and program for visualizing STRUCTURE output and implementing the Evanno method. Conserv Genet Resour.

[CR20] Edae EA, Olivera PD, Jin Y, Poland JA, Rouse MN (2016). Genotype-by-sequencing facilitates genetic mapping of a stem rust resistance locus in *Aegilops umbellulata*, a wild relative of cultivated wheat. BMC Genomics.

[CR21] Endelman JB (2011). Ridge regression and other kernels for genomic selection with R package rrBLUP. Plant Genome.

[CR22] Evanno G, Regnaut S, Goudet J (2005). Detecting the number of clusters of individuals using the software STRUCTURE: a simulation study. Mol Ecol.

[CR23] Feng JY, Wang MN, Chen XM, See DR, Zheng YL (2015). Molecular mapping of *YrSP* and its relationship with other genes for stripe rust resistance in wheat chromosome 2BL. Phytopathology.

[CR24] Flath K, Schulz P, Klocke B (2021) RustWatch – das erste Frühwarnsystem für Getreideroste in Europa. In: Julius Kühn-Institut (Hrsg) 62. Deutsche Pflanzenschutztagung: Gesunde Pflanzen in Verantwortung für unsere Welt. 21.–23. September 2021, Julius-Kühn-Archiv 467, Quedlinburg, pp 211–212

[CR25] Fox J, Weisberg S (2019) An R companion to applied regression, 3rd edn. Sage, Thousand Oaks

[CR26] Fu D, Uauy C, Distelfeld A, Blechl A, Epstein L (2009). A kinase-START gene confers temperature-dependent resistance to wheat stripe rust. Science.

[CR27] Gessese M, Miah H, Bansal U, Bariana H (2021). Genetics of stripe rust resistance in a common wheat landrace Aus27492 and its transfer to modern wheat cultivars. Can J Plant Pathol.

[CR28] Guo Q, Zhang ZJ, Xu YB, Li GH, Feng J (2008). Quantitative trait loci for high-temperature adult-plant and slow-rusting resistance to *Puccinia striiformis* f. sp. *tritici* in wheat cultivars. Phytopathology.

[CR29] Hovmøller MS (2007). Sources of seedling and adult plant resistance to *Puccinia striiformis* f. sp. *tritici* in European wheats. Plant Breed.

[CR30] Hovmøller MS, Sørensen CK, Walter S, Justesen AF (2011). Diversity of *Puccinia striiformis* on cereals and grasses. Annu Rev Phytopathol.

[CR31] Hovmøller MS, Walter S, Bayles RA, Hubbard A, Flath K (2016). Replacement of the European wheat yellow rust population by new races from the centre of diversity in the near-Himalayan region. Plant Pathol.

[CR32] Jia M, Yang L, Zhang W, Rosewarne G, Li J (2020). Genome-wide association analysis of stripe rust resistance in modern Chinese wheat. BMC Plant Biol.

[CR33] Jighly A, Oyiga BC, Makdis F, Nazari K, Youssef O (2015). Genome-wide DArT and SNP scan for QTL associated with resistance to stripe rust (*Puccinia striiformis* f. sp. *tritici*) in elite ICARDA wheat (*Triticum aestivum* L.) germplasm. Theor Appl Genet.

[CR34] Juliana P, Singh RP, Singh PK, Crossa J, Huerta-Espino J (2017). Genomic and pedigree-based prediction for leaf, stem, and stripe rust resistance in wheat. Theor Appl Genet.

[CR35] Kankwatsa P, Singh D, Thomson PC, Ebrahiem M, Babiker EM (2017). Characterization and genome-wide association mapping of resistance to leaf rust, stem rust and stripe rust in a geographically diverse collection of spring wheat landraces. Mol Breed.

[CR36] Klymiuk V, Yaniv E, Huang L, Raats D, Fatiukha A (2018). Cloning of the wheat *Yr15* resistance gene sheds light on the plant tandem kinase pseudo-kinase family. Nat Commun.

[CR37] Krattinger SG, Lagudah ES, Spielmeyer W, Singh RP, Huerta-Espino J (2009). A putative ABC transporter confers durable resistance to multiple fungal pathogens in wheat. Science.

[CR38] Kumar D, Kumar A, Chhokar V, Gangwar OP, Bhardwaj SC (2020). Genome-wide association studies in diverse spring wheat panel for stripe, stem, and leaf rust resistance. Front Plant Sci.

[CR39] Laidig F, Feike T, Hadasch SRD, Klocke B (2021). Breeding progress of disease resistance and impact of disease severity under natural infections in winter wheat variety trials. Theor Appl Genet.

[CR40] Ledesma-Ramírez L, Solís-Moya E, Iturriaga G, Sehgal D, Reyes Valdes MH (2019). GWAS to identify genetic loci for resistance to yellow rust in wheat pre-breeding lines derived from diverse exotic crosses. Front Plant Sci.

[CR41] Ling W, Xian-chun X, You-liang Z, Zheng-yu Z, Hua-zhong Z (2012). QTL mapping for adult-plant resistance to stripe rust in a common wheat RIL population derived from Chuanmai 32/Chuanyu 12. J Integr Agric.

[CR42] Liu W, Frick M, Huel R, Nykiforuk CL, Wang X (2014). The stripe rust resistance gene *Yr10* encodes an evolutionary-conserved and unique CC-NBS-LRR sequence in wheat. Mol Plant.

[CR43] Losert D, Maurer HP, Leiser WL, Würschum T (2017). Defeating the Warrior: genetic architecture of triticale resistance against a novel aggressive yellow rust race. Theor Appl Genet.

[CR44] Lu Y, Wang M, Chen X, See D, Chao S (2014). Mapping of *Yr62* and a small-effect QTL for high-temperature adult-plant resistance to stripe rust in spring wheat PI 192252. Theor Appl Genet.

[CR45] Maccaferri M, Zhang J, Bulli P, Abate Z, Chao S (2015). A genome-wide association study of resistance to stripe rust (*Puccinia striiformis* f. sp. *tritici*) in a worldwide collection of hexaploid spring wheat (*Triticum aestivum* L.). G3.

[CR46] Macer RCF (1966) The formal and monosomic genetic analysis of stripe rust (*Puccinia striiformis*) resistance in wheat. In: Mackey J (ed) Proceedings of the 2nd international wheat genetics symposium, Lund, Sweden. Hereditas Suppl 2:127–142

[CR47] Marchal C, Zhang J, Zhang P, Fenwick P, Steuernagel B (2018). Bed-domain containing immune receptors confer diverse resistance spectra to yellow rust. Nat Plants.

[CR48] McIntosh RA, Dubcovsky J, Rogers WJ, Morris C, Xia XC (2017) Catalogue of gene symbols for wheat. 2017 Supplement. https://shigen.nig.ac.jp/wheat/komugi/genes/macgene/supplement2017.pdf

[CR49] Michelmore RW, Paran I, Kesseli RV (1991). Identification of markers linked to disease-resistance genes by bulked segregant analysis: a rapid method to detect markers in specific genomic regions by using segregating populations. Proc Natl Acad Sci.

[CR50] Miedaner T, Akel W, Flath K, Jacobi A, Taylor M (2019). Molecular tracking of multiple disease resistance in a winter wheat diversity panel. Theor Appl Genet.

[CR51] Moore JW, Herrera-Foessel S, Lan C, Schnippenkoetter W, Ayliffe M (2015). A recently evolved hexose transporter variant confers resistance to multiple pathogens in wheat. Nat Genet.

[CR52] Mu J, Liu L, Liu Y, Wang M, See DR (2020). Genome-wide association study and gene specific markers identified 51 genes or QTL for resistance to stripe rust in U.S. winter wheat cultivars and breeding lines. Front Plant Sci.

[CR53] Muleta KT, Bulli P, Zhang Z, Chen X, Pumphrey M (2017). Unlocking diversity in germplasm collections via genomic selection: a case study based on quantitative adult plant resistance to stripe rust in spring wheat. Plant Genome.

[CR54] Murphy LR, Santra D, Kidwell K, Yan G, Chen X (2009). Linkage maps of wheat stripe rust resistance genes *Yr5* and *Yr15* for use in marker-assisted selection. Crop Sci.

[CR55] Naruoka Y, Garland-Campbell KA, Carter AH (2015). Genome-wide association mapping for stripe rust (*Puccinia striiformis* f. sp. *tritici*) in US Pacific Northwest winter wheat (*Triticum aestivum* L.). Theor Appl Genet.

[CR56] Peterson R, Campbell A, Hannah A (1948). A diagrammatic scale for estimating rust intensity of leaves and stem of cereals. Can J Res Sect C.

[CR57] Plaschke J, Ganal MW, Röder MS (1995). Detection of genetic diversity in closely related bread wheat using microsatellite markers. Theor Appl Genet.

[CR58] Pradhan AK, Kumar S, Singh AK, Budhlakoti N, Mishra DC (2020). Identification of QTLs/defense genes effective at seedling stage against prevailing races of wheat stripe rust in India. Front Genet.

[CR59] Prins R, Pretorius ZA, Bender CM, Lehmensiek A (2011). QTL mapping of stripe, leaf and stem rust resistance genes in a Kariega × Avocet S doubled haploid wheat population. Mol Breed.

[CR60] Pritchard JK, Stephens M, Donnelly P (2000). Inference of population structure using multilocus genotype data. Genetics.

[CR61] R Core Team (2017). R: A language and environment for statistical computing.

[CR62] Ren Y, He Z, Li J, Lillemo M, Wu L (2012). QTL mapping of adult-plant resistance to stripe rust in a population derived from common wheat cultivars Naxos and Shanghai 3/Catbird. Theor Appl Genet.

[CR63] Rollar S, Geyer M, Hartl L, Mohler V, Ordon F (2021). Quantitative trait loci mapping of adult plant and seedling resistance to stripe rust (*Puccinia striiformis* Westend.) in a multiparent advanced generation intercross wheat population. Front Plant Sci.

[CR64] Rosewarne GM, Singh RP, Huerta-Espino J, Herrera-Foessel SA, Forrest KL (2012). Analysis of leaf and stripe rust severities reveals pathotype changes and multiple minor QTLs associated with resistance in an Avocet × Pastor wheat population. Theor Appl Genet.

[CR65] Rosewarne GM, Herrera-Foessel SA, Singh RP, Huerta-Espino J, Lan CX (2013). Quantitative trait loci of stripe rust resistance in wheat. Theor Appl Genet.

[CR66] Singh RP (1992). Genetic association of leaf rust resistance gene *Lr34* with adult plant resistance to stripe rust in bread wheat. Phytopathology.

[CR67] Singh RP, Singh PK, Rutkoski J, Hodson DP, He X (2016). Disease impact on wheat yield potential and prospects of genetic control. Annu Rev Phytopathol.

[CR68] Spielmeyer W, McIntosh RA, Kolmer J, Lagudah ES (2005). Powdery mildew resistance and *Lr34/Yr18* genes for durable resistance to leaf and stripe rust cosegregate at a locus on the short arm of chromosome 7D of wheat. Theor Appl Genet.

[CR69] Sui XX, Wang MN, Chen XM (2009). Molecular mapping of a stripe rust resistance gene in spring wheat cultivar Zak. Phytopathology.

[CR70] Tehseen MM, Tonk FA, Tosun M, Amri A, Sansaloni CP (2021). Genome-wide association study of resistance to *PstS2* and *Warrior* races of *Puccinia striiformis* f. sp. *tritici* (stripe rust) in bread wheat landraces. Plant Genome.

[CR71] Tsomin Y, Wenhua S, Kequan S (1990). Monosomic analyses of stripe rust and leaf rust resistance genes in winter wheat varieties Luquyu and Yantar. Euphytica.

[CR72] Vazquez MD, Peterson CJ, Riera-Lizarazu O, Chen X, Heesacker A (2012). Genetic analysis of adult plant, quantitative resistance to stripe rust in wheat cultivar “Stephens” in multi-environment trials. Theor Appl Genet.

[CR73] Wang S, Wong D, Forrest K, Allen A, Chao S (2014). Characterization of polyploid wheat genomic diversity using a high-density 90,000 single nucleotide polymorphism array. Plant Biotechnol J.

[CR74] Wimmer V, Albrecht T, Auinger HJ, Schön CC (2012). synbreed: a framework for the analysis of genomic prediction data using R. Bioinformatics.

[CR75] Xu Y, Wang J, Crouch JH (2008) Selective genotyping and pooled DNA analysis: an innovative use of an old concept. Recognizing past achievement, meeting future needs. In: Proceedings of the 5th international crop science congress, April 3–18; Jeju, Korea

[CR76] You Q, Zhai K, Yang D, Yang W, Wu J (2016). An E3 ubiquitin ligase-BAG protein module controls plant innate immunity and broad-spectrum disease resistance. Cell Host Microbe.

[CR77] Zetzsche H, Serfling A, Ordon F (2019) Breeding progress in seedling resistance against various races of stripe and leaf rust in European bread wheat. Crop Breed Genet Genom 1:546. 10.20900/cbgg20190021

[CR78] Zhang C, Huang L, Zhang H, Hao Q, Lyu B (2019). An ancestral NB-LRR with duplicated 3′UTRs confers stripe rust resistance in wheat and barley. Nat Commun.

[CR80] Zhu T, Wang L, Rimbert H, Rodriguez JC, Deal KR (2021). Optical maps refine the bread wheat *Triticum aestivum* cv. Chin Spring Genome Assemb Plant J.

