# Supplementary figures and images for: Genome-wide association study and genomic prediction of resistance to stripe rust in current Central and Northern European winter wheat germplasm
Source: Theor Appl Genet. 2022 Aug 26;135(10):3583–95. doi: 10.1007/s00122-022-04202-z (PMC9519682; doi:10.1007/s00122-022-04202-z)

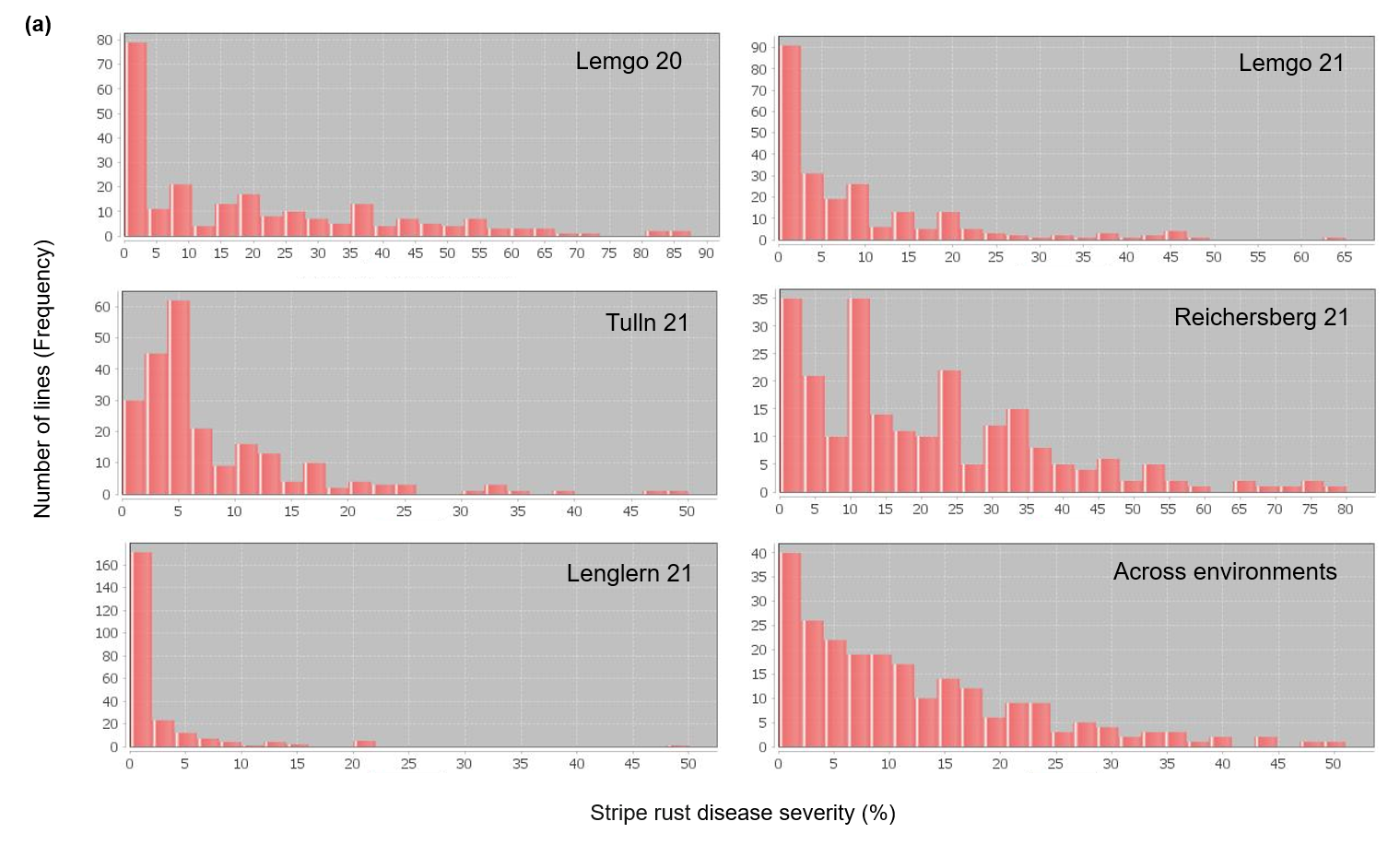

Supplement: Supplementary file 2 — Fig. S1. a) Frequency distribution of stripe rust disease severity (%) in different field trials and across environments.(TIF 900 kb) [file 122_2022_4202_MOESM2_ESM.tif]

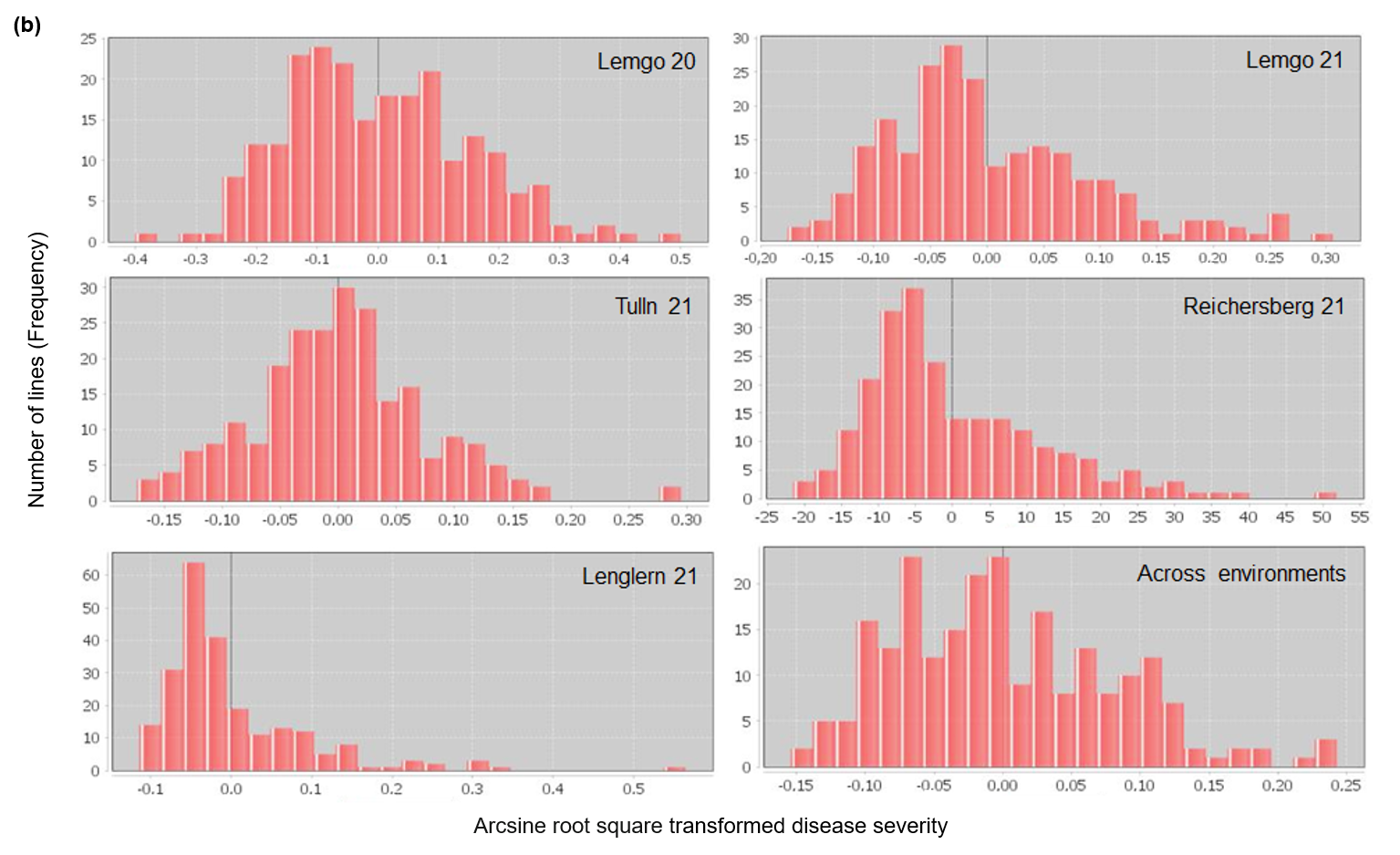

Supplement: Supplementary file 3 — b) Residual frequency distribution of arcsine square root transformed of disease severity in different field trials and across environments. (TIF 1164 kb) [file 122_2022_4202_MOESM3_ESM.tif]

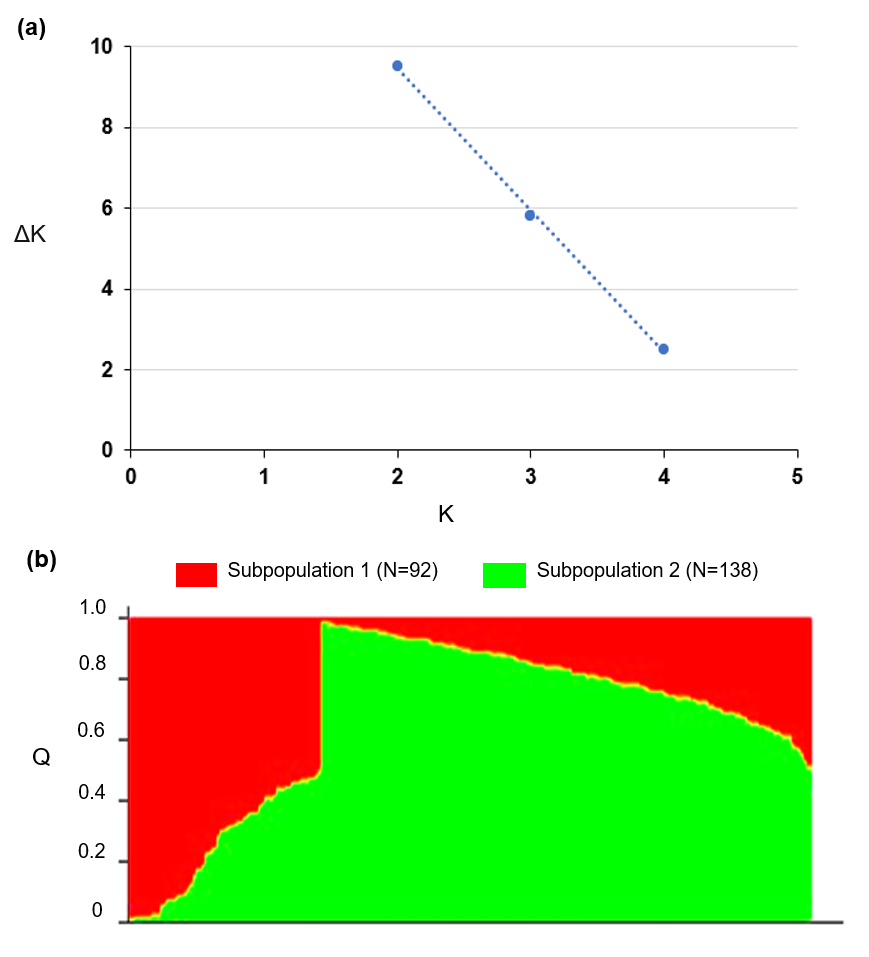

Supplement: Supplementary file 4 — Fig. S2 Population structure among 230 winter wheat cultivars and breeding lines. a) Determination of the number of subpopulations via the ad hoc statistic ΔK. a) Stacked bar plots of ancestry relationship of genotypes based on membership coefficient of individuals (Q). (TIF 88 kb) [file 122_2022_4202_MOESM4_ESM.tif]

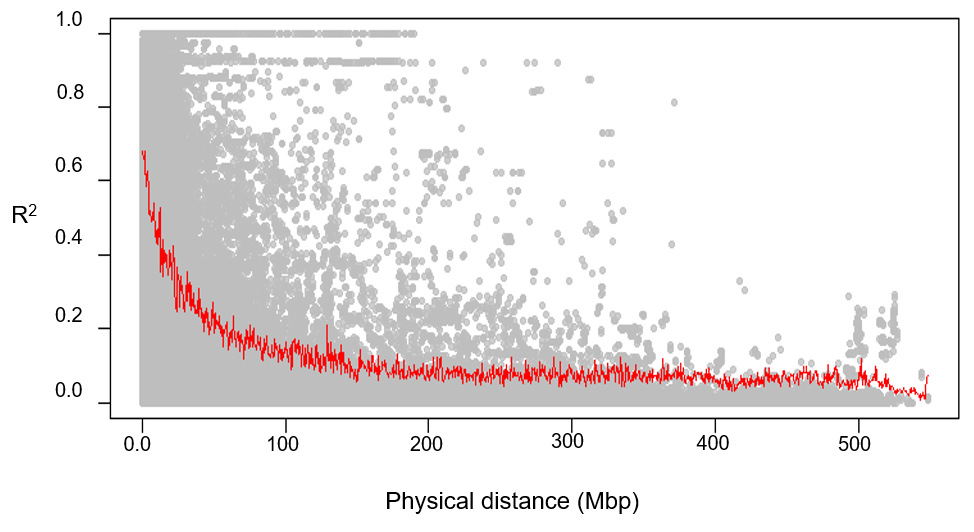

Supplement: Supplementary file 5 — Fig. S3 Scatter plot of linkage disequilibrium (R2) versus inter-marker physical distance (Mbp), with the average R2 of increasing intervals of 0.5 Mbp physical distance plotted as a red line. (TIF 145 kb) [file 122_2022_4202_MOESM5_ESM.tif]

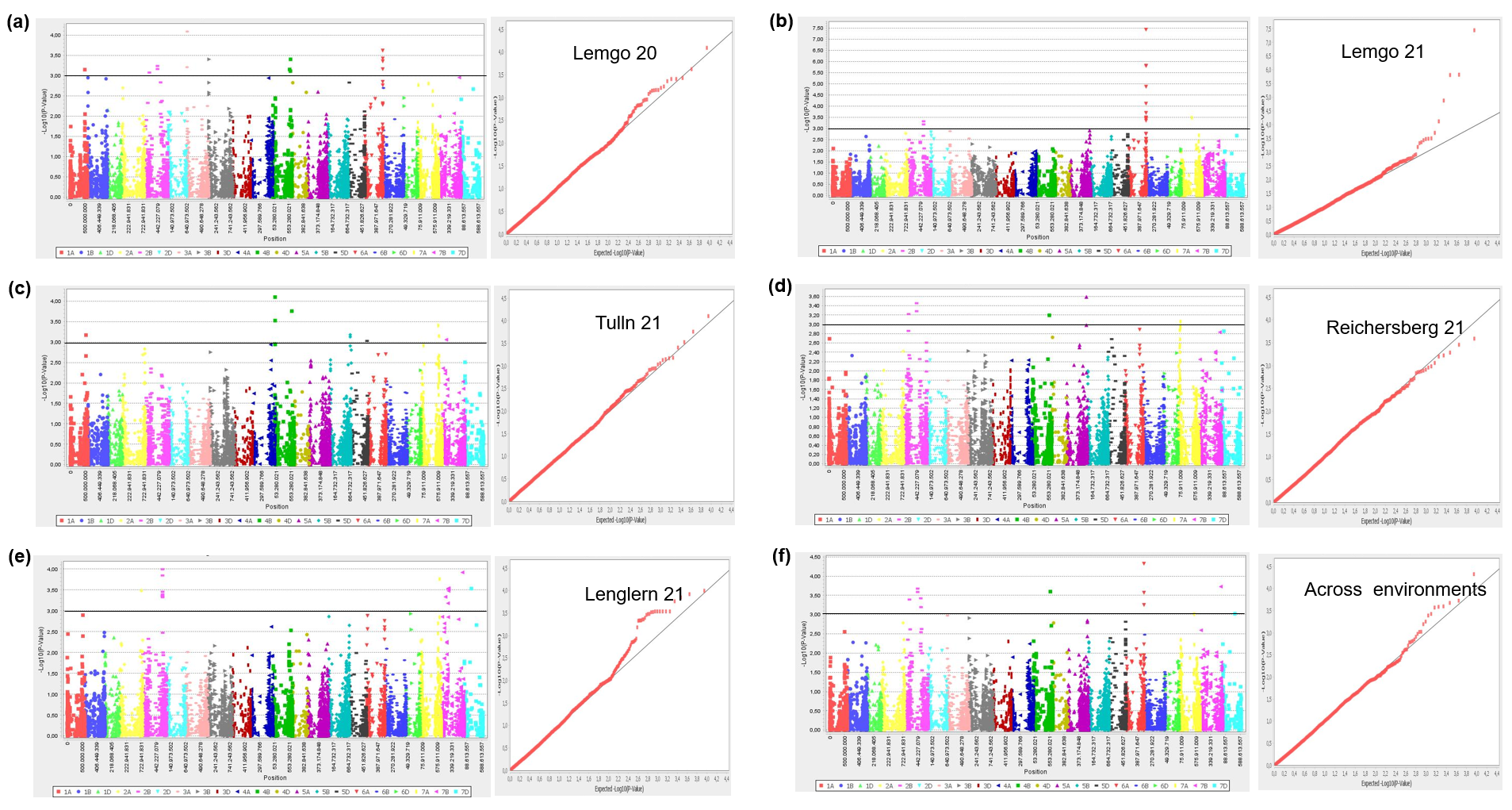

Supplement: Supplementary file 6 — Fig. S4. Manhattan plots showing the association of single nucleotide polymorphisms in the 230 genotypes (left) and quantile–quantile plot comparing the performance of the mixed linear model (right) used in the genome-wide association study for stripe rust resistance in different field trials (a-e) and across environments (f). The horizontal black line represents the genome-wide significance threshold. (TIF 1586 kb) [file 122_2022_4202_MOESM6_ESM.tif]
